# MS-1 *magA*: Revisiting Its Efficacy as a Reporter Gene for MRI

**DOI:** 10.1177/1536012116641533

**Published:** 2016-04-26

**Authors:** Sofia M. Pereira, Steve R. Williams, Patricia Murray, Arthur Taylor

**Affiliations:** 1 Institute of Translational Medicine, University of Liverpool, Crown Street, Liverpool, United Kingdom; 2 Centre for Imaging Sciences, University of Manchester, Oxford Road, Manchester, United Kingdom

**Keywords:** cellular imaging and trafficking, cell tracking, viral vector, magnetic resonance imaging

## Abstract

Bacterial genes involved in the biomineralization of magnetic nanoparticles in magnetotactic bacteria have recently been proposed as reporters for magnetic resonance imaging (MRI). In such systems, the expression of the bacterial genes in mammalian cells purportedly leads to greater concentrations of intracellular iron or the biomineralization of iron oxides, thus leading to an enhancement in relaxation rate that is detectable via MRI. Here, we show that the constitutive expression of the *magA* gene from *Magnetospirillum magnetotacticum* is tolerated by human embryonic kidney (HEK) cells but induces a strong toxic effect in murine mesenchymal/stromal cells and kidney-derived stem cells, severely restricting its effective use as a reporter gene for stem cells. Although it has been suggested that *magA* is involved in iron transport, when expressed in HEK cells, it does not affect the transcription of endogenous genes related to iron homeostasis. Furthermore, the *magA*-induced enhancement in iron uptake in HEK cells is insignificant, suggesting this gene is a poor reporter even for cell types that can tolerate its expression. We suggest that the use of *magA* for stem cells should be approached with caution, and its efficacy as a reporter gene requires a careful assessment on a cell-by-cell basis.

## Introduction

Over the recent years, there has been a growing interest in the development of reporter genes for the tracking of cells in vivo. The use of reporter genes is now well established in the field of optical imaging, where the use of luciferases has led to a great progress in noninvasive longitudinal imaging.^1,2^ However, the same is not true for magnetic resonance (MR) imaging (MRI), where the use of reporter genes is still in its infancy. Iron oxide nanoparticles (IONPs) have been used for many years as a means to label cells, allowing their imaging in vivo via MRI^3-5^ but suffer from drawbacks such as their dilution upon cell division, the potential degradation in vivo as well as the possibility of being transferred to host cells, leading to false positives. Reporter genes have the potential to overcome some of these drawbacks but at the expense of a much smaller degree of contrast per cell, as iron loading and magnetization are usually much lower than can be achieved with IONPs.^6,7^ The purported advantages that reporter genes can offer, in particular, the lack of false positives and the possibility of using cell-type-specific promoters to allow the monitoring of cell phenotype and differentiation are nevertheless compelling. As yet, however, studies demonstrating the effectiveness of MR reporters in such applications are lacking.

Reporters for MRI usually comprise genes involved with iron homeostasis, where transferrin receptor^7,8^ and ferritin^7,9^ are the most well-studied systems, with the latter being the most popular.^10^ Other genes such as tyrosinase^11^ and β-galactosidase^12^ have also been explored but sometimes require the use of injectable substrates. The use of transferrin receptor or ferritin as reporter genes relies on their overexpression in the target cells, thus stimulating the uptake and storage of iron and therefore increasing the cell’s iron levels. Because the cells expressing the reporters have more iron than host cells, they display a quicker transverse decay rate and generate contrast via a loss of signal in T_2_-weighted images. Most of these genes have been modified so as to allow a maximum capacity for iron accumulation. For instance, iron-responsive elements are often removed to bypass the cell’s own endogenous regulation of iron uptake,^7^ and mitochondrial ferritin has been engineered for cytoplasmic expression and suggested to load iron more efficiently than cytosolic ferritin.^13^ Recently, efforts have also been made toward the use of bacterial genes, in particular those originating from magnetotactic bacteria (MTB), as MRI reporters. Magnetotactic bacteria are aquatic bacteria whose motility is directed by the geomagnetic field lines, a behavior that is made possible due to unique organelles called magnetosomes that contain magnetic crystals. There is a great biodiversity of MTBs that biomineralize crystals of different sizes, morphologies, and chemical composition. Although it is known that the genes responsible for magnetosome formation are usually found in specific clusters within the MTB’s genome, the exact role of each gene and protein in the uptake of iron and subsequent biomineralization of magnetic crystals is still elusive.^14^ An early study^15^ suggested that the gene *magA* from *Magnetospirillum* sp AMB-1 (hereon referred to as AMB-1) could be a membrane iron transporter involved with the uptake of iron in this strain, and a homologous gene was later isolated from *Magnetospirillum magnetotacticum* MS-1^16^ (hereon referred to as MS-1). Because of its putative involvement in iron uptake, *magA* has been proposed as a potential MRI reporter, and although the gene originates from bacteria, some reports suggest this gene can be successfully expressed in mammalian cells.^17^ So far, however, research on the use of *magA* as a reporter gene is limited. The gene isolated from AMB-1 has been explored in 3 reports,^17-19^ all originating from the same institution and involving the imaging of tumor xenografts from human melanoma (MDA-MB-435 cells). The use of MS-1 *magA*, on the other hand, has been reported in 2 studies, one of which involved human embryonic kidney (HEK) cells^20^ and another which involved human embryonic stem cells (ESCs).^21^ In both cases, cells expressing a tetracycline-regulated MS-1 *magA* were injected intracranially and imaged via MRI. Here, we explore whether MS-1 *magA* is a suitable gene for the MR tracking of adult stem cells, with a focus on its potential as a reporter for regenerative medicine therapies.

## Methods

### Cell Culture

Multipotent murine mesenchymal stem/stromal cells (CRL-12424; ATCC, Teddington, United Kingdom), mouse kidney-derived stem cells H6,^22^ and HEK 293 T(N) cells (LV900A-1; System Biosciences, Mountain View, California) were cultured in Dulbecco Modified Eagle Medium (DMEM) containing 10% fetal calf serum (FCS) and 1% l-glutamine at 37°C under a humidified atmosphere with 5% CO_2_. All culture media and supplements were purchased from Sigma-Aldrich, Gillingham, United Kingdom, unless stated otherwise.

### Generation of Lentiviral Constructs and Transduction

The MS-1 *magA* gene was obtained as a gift from Elliot Meyerowitz (plasmid 21751; Addgene, Cambridge, Massachusetts). This gene has been deposited in Addgene and originates from the California Institute of Technology, the institution that first isolated it.^16^ MS-1 *magA* complementary DNA (cDNA) was cloned into the pHIV dTomato lentiviral vector (Addgene plasmid 21374). Sequencing of the resulting plasmid suggests 2 amino acid substitutions (S94L) and (P390S) which have also been identified by the depositor as well as 2 additional silent mutations that do not alter amino acid coding (Supplemental Information). Viral production and titration methods were followed as described previously.^23^ For cell transduction with lentiviral particles, cells (10^3^ cells/well in a 48-well plate) were transduced with a specific multiplicity of infection (MOI) for 16 hours in the presence of polybrene (8 μg/mL). An MOI of 1 was used to evaluate the cell’s tolerance to the transgene and an MOI of 5 to obtain a population of cells efficiently expressing the transgene. Transduction of cells was performed in 3 independent experiments (n = 3). After transduction, cells were allowed to expand for 6 days. After 6 days, the HEK cells were subcultured every 2 to 3 days. Nontransduced cells served as controls and were maintained at the same passage number.

### Flow Cytometry and Fluorescence Microscopy

Expression of dTomato was assessed with flow cytometry using a BD FACScalibur instrument (BD Biosciences, Oxford, United Kingdom), with a 488-nm excitation laser and FL2 detector, and via fluorescence microscopy using a Leica DM IL inverted fluorescence microscope coupled to a Leica DFC420C camera (Leica Microsystems, Milton Keynes, United Kingdom).

### Cell Viability and Immunofluorescence

Cell viability was quantified from 4 hours posttransduction (day 0) up to 6 days posttransduction and normalized to nontransduced cells. Cell viability was measured with the CCK-8 assay (Sigma), which is based on the reduction in a water-soluble tetrazolium salt by cellular dehydrogenases, according to manufacturer’s instructions. The presence of the microtubule-associated protein 1A/1B light chain 3 (LC3), an autophagy marker that reveals the presence of autophagosomes was evaluated via immunofluorescence. Cells (2 × 10^3^ cells/well in a 8-well glass chamber slide) were fixed with 4% formaldehyde, washed, permeabilized with 0.1% Triton-X 100, and blocked for 1 hour with 10% chicken serum. Primary anti-LC3A/B antibody (ab58610; Abcam, Cambridge, United Kingdom, 1:200 dilution) was then incubated along with 1% chicken serum and 0.1% Triton-X 100 for 2 hours. After washing, secondary chicken anti-rabbit antibody was applied for 2 hours. Cell nuclei were stained with 4′,6-diamidino-2-phenylindole. Negative controls were performed, where the protocol was followed as described, with the exclusion of the primary antibody. Micrographs were acquired under epifluorescence illumination using a Leica DM2500 microscope coupled to a Leica DFC420C camera.

### Quantitative Reverse Transcription Polymerase Chain Reaction and Western Blotting

Quantitative reverse transcription polymerase chain reaction (RT-qPCR) and Western blotting (WB) were performed to evaluate whether the expression of iron-regulatory proteins (ferritin heavy chain [FTH1] and transferrin receptor 1 [TFR1]) was affected by transduction with *magA*. For RT-qPCR, 9 × 10^4^ HEK cells were collected, and cDNA synthesis and RT-qPCR (CFX Connect Real-Time PCR Detection System; Bio-Rad, Hemel Hempstead, United Kingdom) were performed according to the manufacturer’s instructions (Fast SYBR Green Cells-to-CT kit; Life Technologies, Paisley, United Kingdom). The TATA box binding protein (*TBP*, NM_003194.4)^24^ and glyceraldehyde 3-phosphate dehydrogenase (*GAPDH*, NM_002046.4) genes were used as reference genes for data normalization. Primers for reference and target transcripts are detailed in the Supplementary Information. Data analysis was performed using the CFX System Test Software (Bio-Rad), using the ΔΔCt^25^ method. For WB, 10^6^ cells were used for protein extraction, and all methodologies and materials used were previously described.^7^ The following antibodies were used for WB: anti- FTH1 (ab65080; Abcam), anti-TFR1 (ab84036; Abcam), and anti-actin (ab1801; Abcam) as primary antibodies and IRDye 680RD Donkey anti-Rabbit (926-68073; Licor, Cambridge, United Kingdom) used as a secondary antibody. Total protein gels were used as a reference for data normalization, and actin was used as a reference protein to confirm data normalization.

### Iron Uptake, Quantification, and Cell Pellet Relaxation Measurements

For iron uptake, HEK cells (5×10^4^ cells/well in a 6-well plate) were plated and cultured in medium supplemented with 50 μmol/L l-ascorbic acid,^26^ 1.28 mmol/L human holo-transferrin, and 0.2 or 2 mmol/L ferric citrate (Fe-cit) for 4 days. For nonsupplemented controls, cells were cultured in regular culture medium. After this period, 10^6^ cells were harvested and used for iron quantification as described elsewhere.^7^ In brief, the procedure involves the digestion of cells in hydrochloric acid and potassium permanganate, followed by colorimetric determination of iron with a ferrozine-based reagent. To obtain a cell pellet for the quantification of T_2_ relaxation time, 10^7^ HEK cells were fixed with 4% formaldehyde, transferred to 0.2 mL polypropylene tubes, and centrifuged for 30 minutes at 13 400×*g*. The supernatant was discarded, and 1% low-melting agarose was used to cover the top of the cell pellet. The MR images were acquired with a 7T Avance III MRI instrument (Bruker, Coventry, United Kingdom) using a 38 mm transmit/receive quadrature volume coil and a modified Rapid Acquisition with Refocused Echoes sequence. Cell pellets were imaged with the following parameters: field of view: 30 × 30 mm; matrix size: 256 × 256 pixels; slice thickness: 1 mm; averages: 1; repetition times (TR): 5000, 3000, 1500, 800, 400, and 200 ms; echo times (TE): 11, 22, 55, 77, and 99 ms; acquisition time: 17 minutes 26 seconds. The T_2_ relaxation times from regions of interested defined by the area of the pellet were obtained with Paravision 5.0 (Bruker) and is based on the time constant of the exponential decay of the T_2_ signal strength. Data are only presented for T_2_, although enhanced T_2_ relaxation was accompanied by enhanced T_1_ recovery.

### Transmission Electron Microscopy

The HEK cells (9×10^4^ cells/3.5 cm dish) were incubated with 0.2 mmol/L Fe-cit for 4 days and then fixed with 4% formaldehyde/2.5% glutaraldehyde, postfixed with 1% osmium tetroxide, dehydrated, and embedded in epoxy resin. Thin sections (70 nm) were then collected over copper grids containing a Formvar support film. Samples were analyzed with an FEI Tecnai G2 Spirit BioTwin microscope (Eindhoven, the Netherlands) operated at 100 kV.

## Results

**Figure 1.**
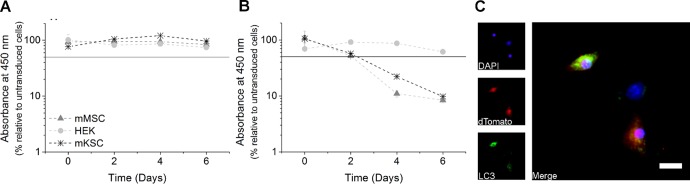
Stem/progenitor cells did not tolerate the expression of MS-1 *magA*. The viability of HEK, mMSC, and mKSC over a course of 6 days after transduction with (A) the empty dTomato vector or (B) magA:dTomato. Viability was quantified using a colorimetric assay based on the water-soluble tetrazolium salt (WST-8) and is expressed as the absorbance at 450 nm in relation to the control (nontransduced) cells. (C) LC3 staining of mKSC suggested the presence of autophagic vesicles. Scale bar represents 20 µm. HEK indicates human embryonic kidney; mMSC, mouse mesenchymal/stromal cells; mKSC, mouse kidney stem/progenitor cells.

Transduction of HEK 293TN cells, mouse mesenchymal/stromal cells (mMSCs), and mouse kidney stem/progenitor cells (mKSCs) with the empty vector (dTomato) or the magA:dTomato construct with an MOI of 1 revealed that although the parent construct was well tolerated by all cell lines (Figure 1A), *magA* was only tolerated by HEK cells. The other 2 cell lines displayed a dramatic loss of viability (Figure 1B), with most cells having died and a few cells having their proliferation arrested after a period of 6 days. Immunostaining for the autophagosome marker, LC3, suggested the presence of autophagic vesicles in mKSC expressing magA:dTomato (Figure 1C), although this was not observed for the other cell lines.

**Figure 2.**
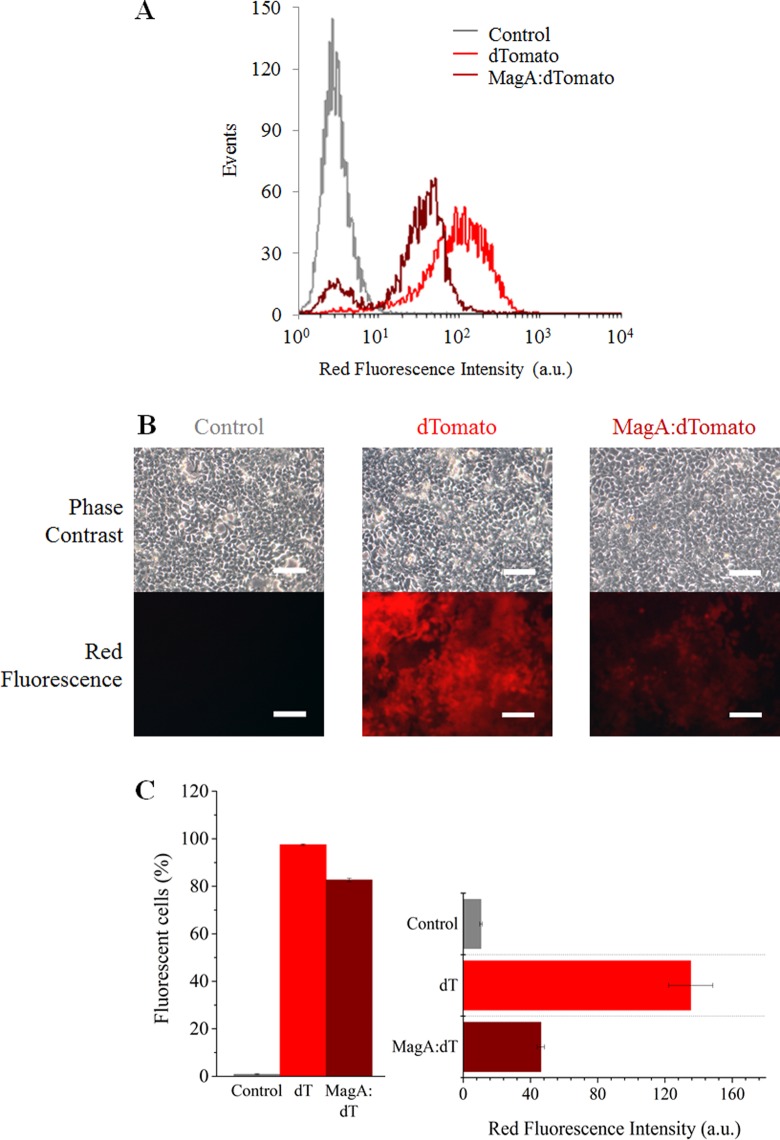
Transduction efficiency of HEK cells is over 80% with both dTomato (dT) and magA:dTomato (magA:dT) lentiviral particles. A, Flow cytometry histogram showed successful integration of the transgenes. B, Phase-contrast and fluorescence images of cells that were transduced with each of the constructs. C, The fraction of positive cells and corresponding red fluorescence intensity as measured via flow cytometry. HEK indicates human embryonic kidney.

To evaluate the effectiveness of *magA* as an MR reporter, HEK cells were transduced with an MOI of 5 to allow for an efficient expression of the gene. Flow cytometry and fluorescence microscopy analysis of the cell populations revealed that over 80% of the cells displayed red fluorescence under these conditions, confirming successful integration (Figure 2). The fluorescence intensity of the magA:dTomato transduced cells was noticeably lower than that of the cells transduced with dTomato alone, which is likely due to the differences in construct size and the fact that dTomato is expressed downstream of an internal ribosome entry site.

**Figure 3.**
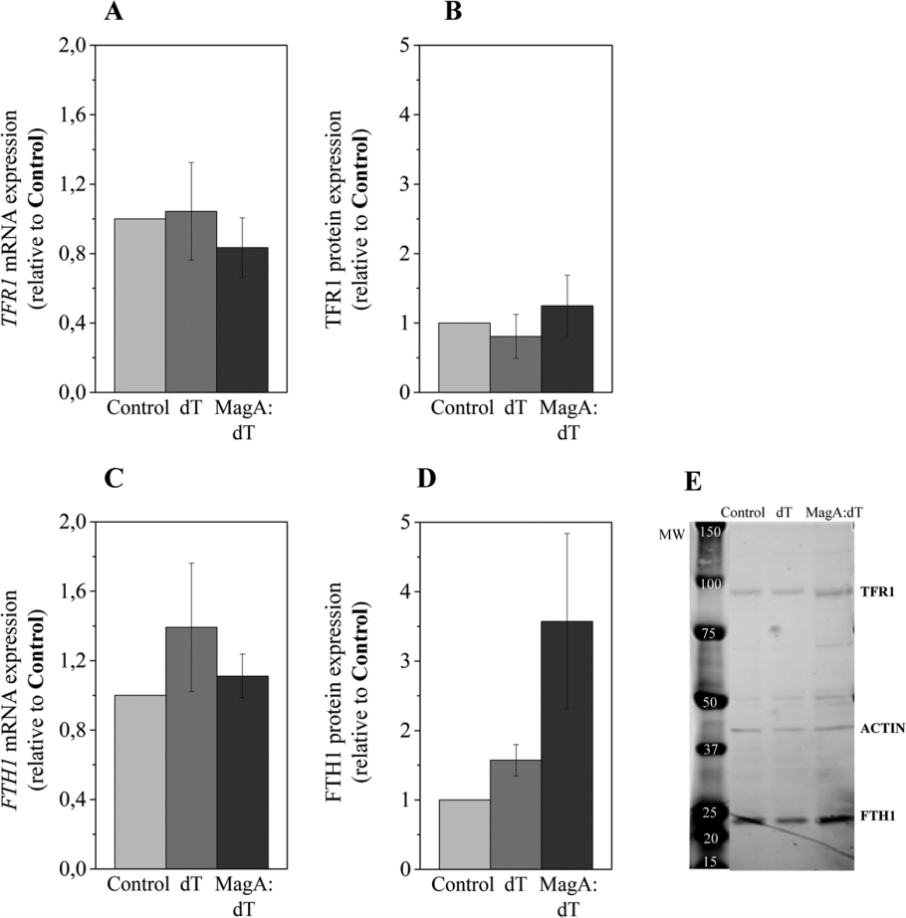
MS-1 magA affects ferritin expression in HEK cells. Relative quantification of (A) *TFR1* transcripts, (B) TFR1 protein, (C) *FTH1* transcripts, and (D) FTH1 protein. Error bars represent SEM (n = 3). (E) Representative Western blot. The molecular weight (MW) ladder and the bands for TFR1, FTH1, and actin are indicated. Control: nontransduced cells; dT: cells transduced with the empty construct (dTomato); magA:dT: cells transduced with the magA:dTomato construct. HEK indicates human embryonic kidney; *TFR1*, transferrin receptor 1; *FTH1*, ferritin heavy chain; SEM, standard error of the mean.

Because *magA* is thought to be involved in iron transport, we have assessed whether its overexpression affects the transcription and translation of *TFR1* and *FTH1*, 2 important genes that regulate iron storage and transport. No differences in the relative transcription or translation of *TFR1* were observed (Figure 3A and B) when cells were transduced with magA:dTomato. Ferritin translation was increased by nearly 4-fold (Figure 3D and representative WB Figure 3E) in respect to controls, with no changes in the relative messenger RNA levels (Figure 3C).

**Figure 4.**
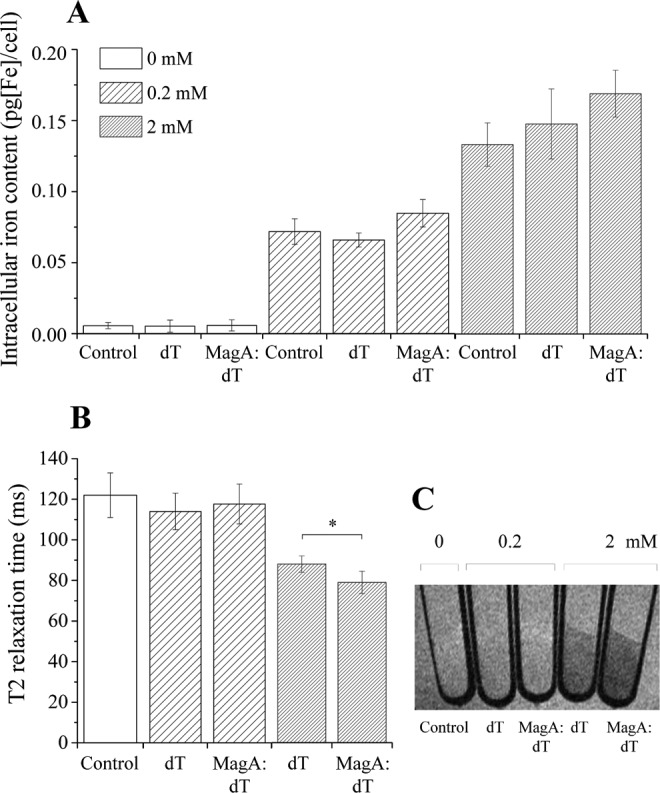
The enhancement in intracellular iron content and relaxometric properties was small for HEK cells transduced with MS-1 magA. (A) Intracellular iron content and (B) T_2_ relaxation of HEK cells cultured for 96 hours in regular medium or supplemented with ascorbic acid (50 µmol/L), human holo-transferrin (1.28 mmol/L), and ferric citrate at a concentration of 0.2 or 2 mmol/L. (C) The MRI of the cell pellets as obtained with a TR of 5000 ms and a TE of 99 ms. Error bars represent SEM (n = 3). Control: nontransduced cells; dT: cells transduced with the empty construct (dTomato); magA:dT: cells transduced with the magA:dTomato construct. HEK indicates human embryonic kidney; MR, magnetic resonance; TR, repetition times; TE, echo times.

The most important aspect of iron-related reporters is their capacity to enhance the iron content of cells, thus leading to contrast in MRI. The intracellular iron content of HEK cells as assessed when cultured in regular culture medium or in the presence of Fe-cit is shown in Figure 4. A gradual increase is seen as a response to iron supplementation, with a 5-fold increment in intracellular iron content when cells were cultured in the presence of 0.2 mmol/L Fe-cit and a further increase to nearly 10-fold when supplemented with 2 mmol/L Fe-cit (Figure 4A). Surprisingly, no statistical differences in relation to controls were found for cells expressing magA:dTomato. When the transversal (T_2_) relaxation time of cell pellets was measured, a good correlation with the iron content was observed (Figure 4B and C) and when supplemented with 2 mmol/L Fe-cit a small but significant difference was seen for cells expressing magA:dTomato with respect to cells expressing dTomato only (*P* = .046).

**Figure 5.**
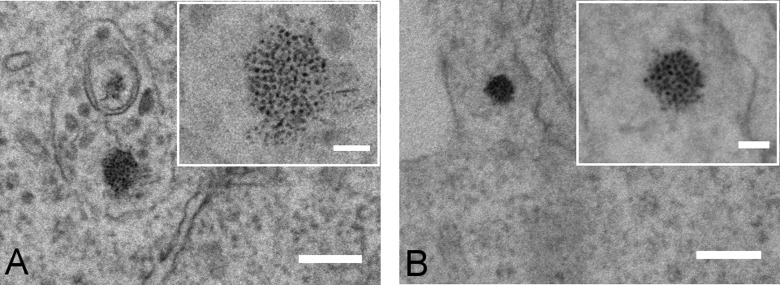
Electron-dense spots could be identified via EM irrespective of the presence of a reporter gene. EM images of (A) dTomato and (B) magA:dTomato transduced cells supplemented with 0.2 mmol/L Fe-cit. Scale bars: 200 nm (50 nm in insets). EM indicates electron microscope. Fe-cit, ferric citrate.

The expression of *magA* in mammalian cells has been previously suggested to direct the formation of nanoparticles similar to those found in magnetosomes.^20^ To confirm if that was the case, we analyzed cells supplemented with Fe-cit via electron microscopy. Electron-dense spots were found in both dTomato and magA:dTomato cells (Figure 5), usually in enclosed vesicles or within mitochondria. In all cases, electron-dense spots seemed spherical in shape, displayed a diameter of approximately 5 nm, and similar electron density.

## Discussion

Accurate assessment of the biodistribution and survival of cells in vivo once administered into a host is one of the roadblocks associated with the clinical translation of stem cell--based regenerative therapies. The use of reporter genes is an appealing strategy to label cells and generate contrast that is dependent on the cell’s viability, thus providing information on the cell’s spatial localization and survival. In MRI, however, robust MR reporters that allow the reliable tracking of cells are still lacking.

Although iron-regulatory proteins have shown the potential to increase the iron content of certain cells,^9^ thus leading to negative contrast in MR imaging, the amount of iron that can be accumulated is still small when compared to what can be achieved with IONPs. In addition to that, the relaxivity of ferritin, the form in which iron is naturally stored in cells, is relatively low when compared to the IONPs,^6^ further limiting the amount of contrast that can be achieved. Magnetosomes, on the other hand, consist of highly crystalline superparamagnetic crystals with relaxivities similar to that of synthetic IONPs.^27^ It is thus not surprising that a great deal of interest was generated when the expression of MS-1 *magA* was suggested to result in the biosynthesis of IONPs in mammalian cells.^20^ We have cloned the same gene into a lentiviral plasmid and produced lentiviral particles that were used to infect HEK cells, mMSCs, and mKSCs. Similar to previous results,^20^ HEK cells tolerated the transgene and were able to proliferate without any changes in viability. The cells of most relevance for regenerative therapies, mMSC and mKSCs, however, displayed clear signs of toxicity after transduction, with proliferation arrest and death within a period of 6 days. This was not observed for the empty construct, confirming that any toxic effects were arising from *magA* expression. Interestingly, this was observed when using a relatively modest MOI of 1, which is much lower than the one we have previously used when transducing mMSCs with *FTH1* or *TFR1*.*^7^* Although a detailed investigation of the possible factors that could lead to the death of stem/progenitor cells has not been carried out here, at least one of the cell lines exhibited expression of the autophagy marker (LC3), suggesting that cell death could be induced by oxidative stress, a well-known modulator of autophagy.^28^ To date, studies involving the in vitro transfection/transduction of cells with *magA* have involved only HEK cells,^20^ cancer cells,^17-19^ and mouse ESCs (mESCs),^21^ and to our knowledge, this is the first report on the expression of *magA* in adult stem/progenitor cells. It is interesting to note that the one study on the expression of *magA* in mESCs^21^ involved an inducible promoter where the gene was not continuously expressed. Importantly, the authors of this study report a decrease in cell proliferation and increase in cytotoxicity when high levels of *magA* expression are induced for 3 days, which was the maximum period of the time investigated in that particular study. Taken together, these findings seem to suggest that stem cell viability might be significantly compromised when *magA* is constitutively expressed, posing significant limitations toward its effectiveness as a reporter gene for cell-based therapies.

Although cell death prevented us from assessing the iron uptake capacity of stem cells expressing *magA*, we have performed further studies with HEK cells in an attempt to elucidate any mechanisms by which this gene can act as an MR reporter. An MOI of 5 resulted in most of the cell population expressing *magA*, as evaluated by flow cytometry via the co-expression of dTomato. The *magA* gene shows homology to membrane cation--proton antiporters^29,30^ and has been suggested to be involved in iron translocation,^29^ although this has recently been challenged.^30^ We have previously shown that overexpression of iron transporters such as *TFR1* can lead to a decrease in the expression of endogenous transferrin receptors and an upregulation of ferritin expression.^31^ In contrast to *TFR1* overexpression, *magA* did not affect the expression of endogenous *TFR1*, although we have seen a modest increase in FTH1 protein. Although it is tempting to suggest that this could be related to *magA*’s function as an iron transporter, leading to more iron uptake and consequently an increase in *FTH1* translation, recent evidence suggests that *magA* is not only unnecessary for magnetosome formation in MTBs but also unlikely to work as an iron transporter.^30^ It is now known that iron is not the only regulator of ferritin translation and that other signals, including oxidative stress, can lead to increases in ferritin protein.^32^ Considering our observations with stem cells, which did not tolerate the expression of *magA*, it is reasonable to suggest that *magA* expression in HEK cells might also lead to changes in homeostasis with one of the consequences being an increase in *FTH1* translation. This is consistent with the negligible increases in intracellular iron content, which were not significantly higher for cells expressing the transgene. When supplemented with 0.2 mmol/L Fe-cit, we observed an intracellular iron content of approximately 0.075 pg/cell, which is in close agreement to that previously seen for HEK cells supplemented with this concentration of iron.^20^ However, while this previous report suggested up to a 14-fold increase in intracellular iron when *magA* is expressed, this was not seen in the current study. Although a significant reduction in T_2_ relaxation was observed when cells were supplemented with 2 mmol/L Fe-cit, this reduction was extremely small and unlikely to be appreciated in vivo, where conditions are not as homogeneous as in the case of a compressed cell pellet in a test tube. In fact, supplementation of culture medium with Fe-cit was shown to be a much more effective means of increasing the intracellular iron content and T_2_ relaxation rates than the expression of *magA*.

Finally, we investigated whether *magA* expression could lead to the formation of magnetic nanoparticles within the HEK cells. The presence of electron-dense spots in HEK cells expressing *magA* has previously been reported as evidence of IONP biogenesis.^20^ This is certainly one of the most striking observations on the use of *magA* as a reporter, as it suggests that this gene would provide all the machinery necessary for the biosynthesis of magnetic crystals in mammalian cells. The simple observation of electron-dense spots via electron microscopy, however, is not enough to support the presence of crystalline IONPs, which requires other techniques such as Mössbauer spectroscopy for accurate phase identification. It is well accepted that iron-overloaded tissues can display iron-rich ferritin structures of about 6 nm diameter that can be found in clusters and coalescence in membrane-bound bodies (siderosomes).^33^ In fact, animal studies from the mid-1950s have shown that iron overloading leads to the formation of hemosiderin granules consisting of particles of 5 to 6 nm in diameter that can be observed via transmission electron microscopy.^34^ Such granules can also be found in cells loaded with Fe-cit in vitro, even at a concentration of 0.1 mmol/L,^35^ which is lower than we have used here. Thus, it is likely that the high iron supplementation used in studies where the purpose is to achieve maximum MR contrast might lead to the formation of such structures, which we have also seen here irrespective of *magA* expression.

Our results show that the adoption of *magA* as a reporter gene must be approached with caution, particularly when using cells that are of relevance for regenerative therapies. A careful evaluation of the cells’ tolerance to *magA* expression is probably required on a cell-by-cell basis. The only study published so far that did not involve malignant or transformed cells,^21^ which can have a different metabolism to healthy cells, suggests that this gene can be toxic. Furthermore, although cancer cells might tolerate its expression, care must be taken to ensure it does not destabilize cell homeostasis. In fact, the increased iron uptake seen for cells expressing *magA* has been previously suggested to be a stress response to the transgene, rather than the gene functioning as an iron transporter.^30^ Finally, the observation of electron-dense spots in cells overexpressing *magA* must be evaluated with care, as those are likely to consist of ferritin aggregates rather than magnetite crystals.

## Supplementary Material

Supplementary material
