# Supplementary material for: MS-1 magA: Revisiting Its Efficacy as a Reporter Gene for MRI
Source: Mol Imaging. 2016 Apr 26;15:1536012116641533. doi: 10.1177/1536012116641533 (PMC5470133; doi:10.1177/1536012116641533)
Supplement: Supplementary material [file Supplemental_533.pdf]

Supplemental Information

ATGGACCTGCATCATCCCGAACTGACCTATGCCGCCATCGTCGCCCTGGCCGCCGTTCTGTG
CGGCGGGCTGATGACGCGCCTCAAGCAACCGGCCATCGTCGGCTATATCCTGGCCGGGGTGG
TGCTGGGCCCCAGCGGCTTCGGGCTGGTGAGCAACCGCGACGCCGTGGCCACCCTGGCCGAG
TTCGGCGTGCTGATGCTGCTCTTCGTCATCGGCATGAAGCTGGACATCATCCGTTTTCTCGA
AGTGTGGAAGACGGCGGTCTTCACCACCGTTTTGCAGATCGCGGGCTCCATCGGCACGGCGC
TGCTGCTGCGCCACGGTTTGGGCTGGAACCTGGGGCTGGCGGTGGTGCTGGGCTGCGCCGTG
GCGGTGTCGGTCCACCGCCGTGGTGATCAAGGTGCTGGAATCCTCGGGCGAGTTGGAAACGCC
GGTGGGGCGCACCACGCTGGGCATCCTGATTGCCCAGGACATGGCGGTGGTCCCCATGATGC
TGGTGCTGGAATCCTTCGAGACCAAGGTGTTGCTGCCCGCCGATCTGGCCCGCGTGATCCTG
TCCGTGCTGTTCCTGGTGCTGCTGTTCTGGTGGCTGTCCAAGCGCCGTATCGACCTGCCGCT
GACCTCCCGGCTGTCGCGGGATTCCGATCTGGCCACCCTTTCCTCCCTGGCCTGGTGCTTTG
GCACGGCGGCCATTTCCGGGGTGCTGGACCTGTCGCCCGCCTATGGCGCCTTTCTGGGCGGC
GTGGTGCTGGGCAATTCCGCCCAGCGCGACATGCTCTTGAAGCGCGCCCAGCCCATCGGCAG
CGTGCTGTTGATGGTGTTCTTCCTGTCCATCGGGTTGCTGCTTGATTTCAAGTTCATCTGGA
AGAATCTGGGCACCGTTCTCACTCTTTTAGCCATGGTGACGCTGTTCAAGACGGCGCTGAAC
GTCGCCGCGCTGCGTCTGGCGCGTCAGGACTGGCCCAGCGCTTTCCTGGCCGGGGTGGCCCT
GGCCCAGATCGGCGAGTTCTCGTTTTTGCTGGCCGATACCGGCAAGGCGGTTAAGCTGATCA
GCGCCCAGGAGACCAAGCTGGTGGTGGCGGTCACCGTGCTGTCCCTGGTGTTGTCGCCCTTC
TGGCTGTTCACCATGCGGCGTATGCATCGGGTGGCGGCGGTGCATGTCCATTCGTTCCGCGA
ATTGGTCTCGCGGCTTTACGGCGACGAAGCCCGCACCGCGCGGCGGGCCCGCACCCTGGTGC
GGCGGGGCTCTTGGAGGGATGATCACAATGCCGGACCTGGCACTGGAATCTGA

MS-1 magA cDNA sequence. Nucleotides marked in red correspond to two missense mutations reported by the depositor in Addgene, which led to amino acid substitutions S94L and P390S. These substitutions are not conservative and its influence on magA's protein translation is currently unknown. Two other point mutations, which are not reported in Addgene, were found and are marked in green. C is a mismatch from G-C and G is a new insertion. These mutations are silent and do not affect the amino acid coding sequence.

Primer Pairs used for qRT-PCR

**Primer**	**Forward Primer (5'-3')**	**Reverse Primer (5'-3')**	**Product (bp)**
Transferrin Receptor-1 (*TFR1*)	TGAGTGGCTACCTGGGCTAT	CTCCTCCGTTTCAGCCAG TT	74
Ferritin heavy chain-1 (*FTH1*)	TGAGGAGAGGGAGCATGCCG A	CCAGTCATCACGGTCTGG TTT	100
TATA box Binding Protein (TBP)	GACGAGTTCCAGCGCAAGGG TT	GCACCCTGAGGGGAGGC CAA	110
Glyceraldehyde 3-phosphate dehydrogenase (GAPDH)	CGGAAGGCCATGCCAGTGAG C	TGCCAAGGCTGTGGGCAA GG	63

1
